# Refractive and Quality of Vision Outcomes with Toric IOL Implantation in Low Astigmatism

**DOI:** 10.1155/2016/5424713

**Published:** 2016-12-14

**Authors:** Eduardo Scaldini Buscacio, Lia Florim Patrão, Haroldo Vieira de Moraes

**Affiliations:** ^1^Federal University of Rio de Janeiro, Rio de Janeiro, RJ, Brazil; ^2^Hospital de Olhos Niterói, Rio de Janeiro, RJ, Brazil

## Abstract

*Purpose.* To evaluate the refractive and the quality of vision outcomes of toric IOL implantation in patients with low astigmatism.* Design.* Prospective study of single-arm.* Methods.* Patients with corneal astigmatism range from 0,75 D to 1,5 D and cataract that underwent cataract surgery with toric IOL. The measurements were performed preoperatively and 6 weeks after the surgery. Patients were evaluated for visual acuity with and without correction, contrast sensitivity, static and dynamic refraction, and quality of life questionnaire. Pre- and postoperative values were compared and their variations were evaluated for linear correlation.* Results.* 21 eyes of 21 patients. Postoperative mean uncorrected visual acuity was 0.80 ± 0.19, and the best corrected visual acuity was 0.97 ± 0.15. *p* < 0.001 compared to preoperative values. The average postoperative refractive cylinder was −0.34 ± 0.39. The questionnaire's total value before and after surgery was, respectively, 43.20 ± 15.76 and 79.70 ± 10.11 (*p* < 0.001). The correlation coefficients between the values of the questionnaire variation and the UCVA, BCVA, and CS variation were, respectively, 0.548 (*p* = 0.005), 0.508 (*p* = 0.009), and 0.409 (*p* = 0.033).* Conclusion.* Patients with low astigmatism who underwent phacoemulsification with toric IOL implantation experienced significant decrease in refractive astigmatism and improvement in their quality of life.

## 1. Introduction

Freedom from glasses is an increasingly important objective in cataract surgery. Current biometry techniques are precise in the correction of spherical refractive errors (myopia and hyperopia). However, a failure to correct refractive errors associated with astigmatism during cataract surgery may compromise the patient's ability to be free of glasses.

The prevalence of corneal astigmatism is 95% in the population. Recent studies on different ethnic groups have confirmed that among cataract patients; approximately 60% present a prevalence of corneal astigmatism lower than 1.5 diopters (D) and greater than 0.75 D [1, 2].

Among the treatment options for astigmatism, the toric intraocular lens (IOL) implantation is considered to be the most effective. Despite the excellent refractive results for toric lenses, which provide a visual acuity greater than 20/40 in more than 80% of cases and freedom from glasses in approximately 70% of cases [3], residual refractive astigmatism and complaints regarding vision still persist after surgery.

Questionnaires have been developed to evaluate visual quality. One important instrument is the 25-item Visual Functioning Questionnaire (VFQ-25) produced by the National Eye Institute (NEI) of the United States. The questionnaire measures the influence of low vision and visual symptoms on specific areas of overall health, such as the emotional state and social functioning [4].

The objective of this study was to evaluate the refractive and the quality of vision outcomes of toric IOL implantation in patients with low astigmatism.

## 2. Methods

### 2.1. Study Design and Sample Selection

A single-arm, blinded, prospective study was performed. It included patients with cataracts who repeatedly visited a public hospital and underwent phacoemulsification, preoperative testing, and postoperative testing to measure improvements in distance best corrected (BCVA) and uncorrected (UCVA) visual acuity and contrast sensitivity (CS). The cataract classification, demographic and epidemiological data, and the visual quality questionnaire were performed.

The inclusion criteria were cataract diagnosis confirmed by biomicroscopy, corneal astigmatism range from 0.75 D to 1.5 D, and no previous ocular surgeries or ocular diseases that could compromise vision. The exclusion criteria were incomplete postoperative follow-up and intraoperative and postoperative complications like posterior capsule rupture and IOL misalignment.

Ethics committee approval for the study protocol was obtained. All patients signed an informed consent from before preoperative examinations.

The participants were evaluated before and after phacoemulsification. The following data were used in the study:

- UCVA and BCVA.
- CS.
- Refraction and spherical equivalent (SE).
- Score on the visual quality questionnaire.

The eye with the lower visual acuity was chosen in each patient.

### 2.2. Sample Size

This study was planned to identify a difference between questionnaire domains of at least 20 points, a level of significance of 5%, and 80% power. The standard deviation of a previous study was found to be 20 points, and this was therefore considered the minimal difference for the present study. The sample size was calculated after considering quantitative differences in the group before and after the intervention; accordingly, a minimum of 21 group participants were determined [5].

### 2.3. The Procedure

The same surgeon (E.S.B.) performed all surgeries. The surgery performed was phacoemulsification with a toric IOL implantation while the patient was under topical anesthesia. The main incision was a 2.5 mm clear corneal incision and it was made at 120°.

### 2.4. IOL Specification

Rayner T Flex® IOLs were used in this study. This is an aspheric toric IOL, the IOL edges are squared, and its spherical aberration is neutral. Rayner T Flex has an optic body diameter of 5.75 or 6.25 mm and an overall length of 12 or 12.5 mm. The estimated A-constant is 118.9 and the spherical power ranges from +6 to +30 D and the cylinder power ranges from +1 to +6 D.

### 2.5. IOL Calculation

The data obtained by the IOL Master® 500 were inserted into the supplier's website (RaynerCalculator®), and the intraocular lens that came closest to emmetropia was chosen. The surgically induced astigmatism was set at 0.3 D on the axis where the main incision was made.

### 2.6. Ophthalmologic Evaluation

The ophthalmologic evaluation was performed by a specialist (L.F.P.) and included the patient's clinical history and the following exams: BCVA, UCVA, CS, tonometry, corneal topography, and retinal evaluation.

Biomicroscopy was performed to characterize cataracts by type (cortical, nuclear, or posterior subcapsular) and by grade (1 to 6) according to the international lens opacity classification system (LOCS III) [6].

The participants were evaluated on the first and seventh postoperative days, as well as 6 weeks after surgery.

### 2.7. IOL Stability

The rotational stability of the IOL was evaluated 6 weeks postoperatively at the slit-lamp.

### 2.8. Improved Corrected and Uncorrected Distance Visual Acuity

Visual acuity at 6 meters was measured using the Snellen chart; values were obtained up to the decimal notation.

### 2.9. Contrast Sensitivity

This was measured using the Pelli–Robson chart. As recommended in the chart, the test was performed in a room with uniform lighting, and the table presented a mean luminance of 85 cd/m^2^. This situation was considered photopic, with an acceptable range of 60 to 120 cd/m^2^. This value was tested before the exam using a Gossen-Starlite meter. The patient was seated 1 meter from the table, thus allowing legibility of the optotypes, with a spatial frequency of approximately 1 cycle per degree (CPD) at this testing distance. The CS value considered was that which corresponded to the last group of three letters in which the patient was able to read at least two correctly.

### 2.10. Corneal Astigmatism

Corneal astigmatism was evaluated using the IOL Master 500 device and 6 central points that were 2.5 mm in diameter.

### 2.11. Refractive Value

Autorefraction was performed using the Huvitz HRK-7000® device with three measurements from which the mean value was calculated. This value was tested subjectively using the Bausch & Lomb® refractor.

### 2.12. Spherical Equivalent Calculation

The SE was calculated as the sum of the value of the spherical degree and half of the cylindrical degree.

### 2.13. Questionnaire

The NEI VFQ-25 was used. The questionnaire is composed of 25 questions that evaluate various domains of quality of life and visual functioning, as outlined in Table 1 [4].

The patients themselves completed the questionnaires; they were aided by the specialist when questions arose.

Each question corresponds to a numerical value established so that a score of 100 represents the best conditions and a score of 0 reflects the worst conditions. The means were calculated according to the following equation: the mean is defined as the sum of the numerical values of each question within a given domain divided by the number of items evaluated in that domain [4].

### 2.14. Statistical Analyses

The SPSS program, version 23.0 for Mac (SPSS Inc., Chicago, Illinois, USA), was used in the statistical analyses. The normality of the sample was evaluated using the Kolmogorov-Smirnov test. Noncontinuous variables were expressed as frequency, and the continuous variables were expressed as means and standard deviations. The variables were statistically analyzed using the paired *t*-test when the values were continuous and presented normal distribution and using the Wilcoxon signed-rank test when they did not present normal distribution. The Pearson correlation coefficient (for normal distribution variables) and the Spearman correlation coefficient (for variables that did not present normal distribution) were used to compare VFQ with visual acuity, CS, and refractive values. Results were considered significant when *p* < 0.05.

## 3. Results

### 3.1. Clinical and Demographic Characteristics

A total of 21 eyes from 21 patients were studied.

The mean age was 68.9 ± 10 (SD) years and age ranged from 50 to 87 years. Of these participants, 13 (61.9%) were female and 8 (38.1) were male. Fifteen participants (71.4%) had their right eye operated and 6 participants (28.6%) had their left eye operated.

**Figure 1:**
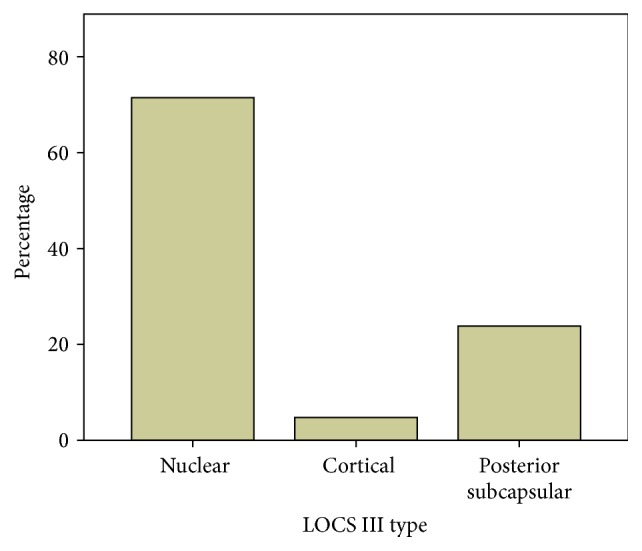
Histogram classifying the type of cataract according to the LOCS III system; this figure shows that 15 (71.4%) were nuclear, 1 (4.8%) was cortical, and 5 (23.8%) were posterior subcapsular.

**Figure 2:**
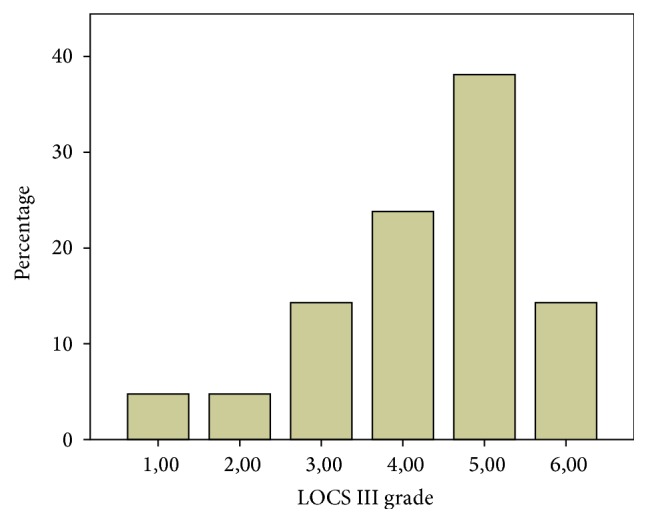
Histogram classifying the type of cataract according to the LOCS III system; this figure shows that 1 (4.8%) was of grade 1, 1 (4.8%) was of grade 2, 3 (14.3%) were of grade 3, 5 (23.8%) were of grade 4, 8 (38.1%) were of grade 5, and 3 (14.3%) of were grade 6.

The results of cataract type as classified by the LOCS III were shown in Figures 1 and 2.

### 3.2. Visual Acuity Results

**Figure 3:**
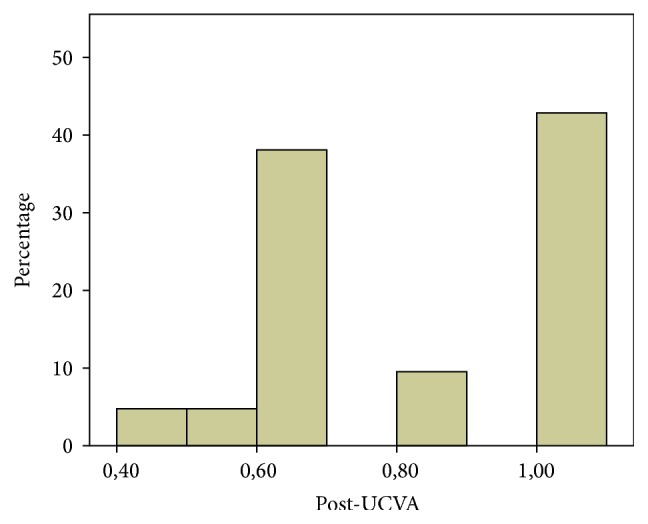
Histogram showing the distribution of postoperative UCVA: 86% of the participants exhibited vision acuity of 0.6 or higher.

As shown in Table 2, the mean preoperative UCVA was 0.2 ± 0.17 (SD) and average postoperative UCVA was 0.80 ± 0.19 (SD); the range of means was 0.60, and the *p* value was less than 0.001 between preoperative and postoperative values in the paired *t*-test. With regard to postoperative UCVA, 47% of patients exhibited visual acuity better than or equal to 20/25, and 86% presented visual acuity better than or equal to 20/30 (Figure 3).

The mean preoperative and postoperative BCVA were 0.28 ± 0.15 (SD) and 0.97 ± 0.15 (SD) respectively, range of means was 0.69, and the *p* value was less than 0.001 between preoperative and postoperative values in the Wilcoxon signed-rank test. With regard to postoperative BCVA, 100% of patients presented visual acuity that was better than or equal to 20/25.

The mean BCVA in the contralateral eye was 0.28 ± 0.25 (SD). When the paired *t*-test was applied, no significant difference in BCVA was found between the eye included in the study and the contralateral eye (*p* = 0.1053).

The mean preoperative and postoperative photopic CS were 1.22 ± 0.32 (SD) and 1.74 ± 0.19 (SD), respectively, range of means was 0.52, and the *p* value was less than 0.001 between preoperative and postoperative values in the paired *t*-test.

### 3.3. Refraction Results

The mean preoperative corneal cylinder (IOL Master) was 1.06 ± 0.27 D, ranged from 0.75 to 1.46 D. Of these, 9.5% were oblique astigmatisms, 28.5% were with-the-rule astigmatisms, and 62% were against-the-rule astigmatisms. The mean topography astigmatism was 1.04 ± 0.3 D, ranged from 0.46 to 1.48 D.

**Figure 4:**
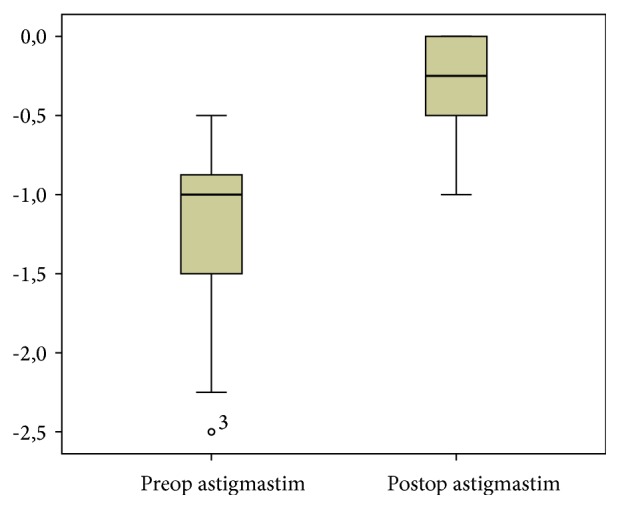
Refractive cylinder boxplot showing statistically significant variation between preoperative and postoperative refractive cylinder values. Preoperative mean was −1.23 D and postoperative mean was −0.34 D. ∘ is the outlier and 3 represents the subject number 3.

**Figure 5:**
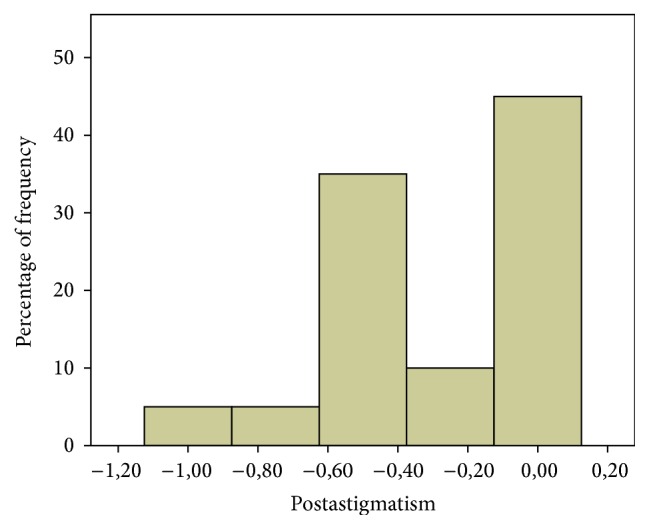
Histogram of postoperative refractive cylinder showing that 86% of the patients presented a postoperative refractive cylinder lower than −0.5 D.

The mean preoperative and postoperative refractive astigmatism were −1.23 ± 0.53 D and 0.34 ± 0.39 D, respectively (Figure 4). Among the participants, 86% presented a postoperative refractive cylinder lower than or equal to −0.5 D, and 52% of participants exhibited a refractive cylinder lower than or equal to −0.25 D (Figure 5). Results of astigmatism and spherical equivalent are presented in Table 3.

### 3.4. Visual Functioning Questionnaire Results

The questionnaire results are presented in Table 4. None of the participants received points for the section on driving.

### 3.5. Relationship between Questionnaire Results and Visual Acuity

**Figure 6:**
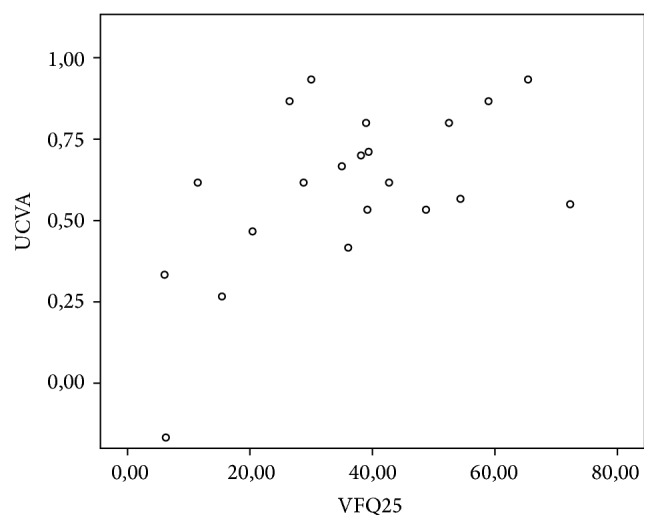
Scatterplot showing the significant correlation between UCVA and total questionnaire score as per the Pearson's coefficient of 0.548 (*p* = 0.005).

**Figure 7:**
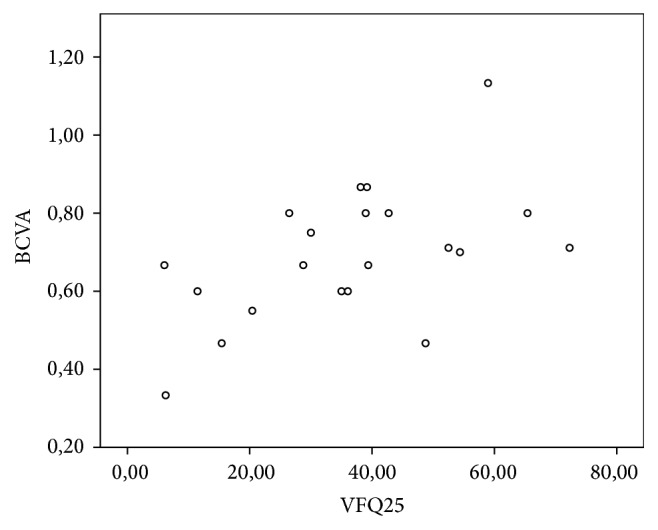
Scatterplot showing the significant correlation between BCVA and total questionnaire score as per the Spearman coefficient of 0.508 (*p* = 0.009).

**Figure 8:**
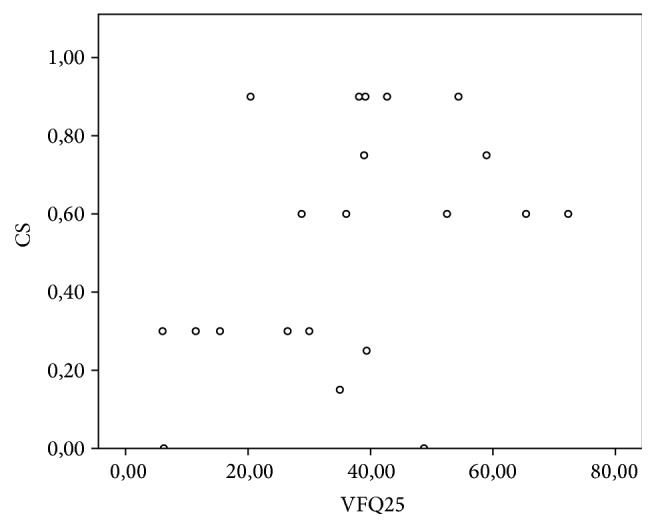
Scatterplot showing the significant correlation between CS and total questionnaire score as per the Pearson coefficient of 0.409 (*p* = 0.033).

A significant linear correlation was observed between the variation in UCVA, BCVA, and CS and variation in VFQ results (Table 5), as shown in the scatterplots (Figures 6, 7, and 8).

## 4. Discussion

Phacoemulsification currently has a refractive function in addition to simple cataract removal. It is important to note that an astigmatism greater than 0.5 D is found in approximately 80% of the cataract patient population [1, 2]; its treatment is therefore fundamental. According to Visser et al., toric IOL implantation is currently considered the most effective method of treatment [3]. Despite the success of modern cataract surgery techniques and their adequate refractive results, patients report many complaints regarding their eyesight, particularly in terms of visual quality (such as halos and blurriness), which lead to decreased quality of life among these patients [7]. Thus, the current study sought not only to present visual and refractive results on a toric IOL implantation in patients with low astigmatism but also to correlate these results with the quality of vision according to a visual functioning questionnaire.

The study sample was composed of 61.9% females and 38.1% males, and the mean patient age was 68.9 years. This sample was similar to those in other studies that have evaluated cataracts in the elderly [1, 2]. No statistically significant difference was observed between UCVA of the studied eye and that of the contralateral eye; thus, the impact of cataract surgery on the quality of life was not affected by disproportionate visual acuity.

With regard to cataract classification, most patients exhibited nuclear cataracts (71.4%), followed by posterior subcapsular cataracts (23.8%) and cortical cataracts (4.8%). According to Prokofyeva et al., the risk factors for nuclear cataracts are an unbalanced diet and poor socioeconomic conditions. This finding explains the higher prevalence of nuclear cataracts among the participants in the present study, which was performed in a developing country [8]. The cataract grades exhibited by the sample were the following: 76.2% of patients were of grades 4, 5, and 6. This proportion of advanced cataracts was higher than those reported by previous studies. Indaram et al. presented a study in which 16.4% of patients had cataracts of grade 4 or higher [9]; this difference may be explained by the socioeconomic conditions and limited access to healthcare among developing country populations.

UCVA is an important parameter when evaluating the refractive effectiveness of cataract surgery. In the present study, the mean postoperative UCVA was 0.8, with 47% of patients experiencing vision that was better than or equal to 0.8 and 96% experiencing vision that was better than or equal to 0.5. Recent reviews of the literature on the surgical outcomes of toric IOLs have presented results in which UCVA greater than or equal to 0.8 was observed in 23% to 100% of cases and in which UCVA greater than or equal to 0.5 was observed in 68% to 100% of cases [3, 10]. Statistically significant variation between preoperative and postoperative values (*p* < 0.001) was also found by Agresta et al. [11].

In this study, the mean postoperative BCVA was 0.97, with 100% of patients exhibiting BCVA greater than 0.8. Preoperative and postoperative variations were found to be statistically significant (*p* < 0.001). In addition, a statistically significant variation was obtained (*p* < 0.001) in the evaluation of CS, with a mean value of 1.74 dB. Similar mean values were observed under similar photopic conditions with low spatial frequency [10, 12].

An important result of the present study was the sample's mean corneal and refractive astigmatisms of −1.06 ± 0.27 D and −1.23 ± 0.53 D, respectively, in the preoperative period and a mean postoperative refractive astigmatism of −0.34 ± 0.39 D. Leon et al. studied a population with low astigmatism, and the preoperative and postoperative refractive astigmatism values were −1.59 ± 0.52 D and −0.40 ± 0.20 D, respectively. Their results were therefore similar to those of the present study. In their study, the postoperative refractive cylinder was lower than or equal to −0.5 D in 86% of patients and lower than or equal to −0.25 D in 52%. Previous studies have reported 25% to 100% patients with a postoperative refractive cylinder lower than or equal to 0.5 D [3]. Another study by Mendicute et al. obtained a preoperative and postoperative refractive astigmatism of −1.75 ± 0.71 D and −0.62 ± 0.46 D, respectively [10].

The mean spherical equivalent values increased without statistical significance (from −0.17 ± 2.33 D to −0.52 ± 0.35 D); however, an important decrease in the standard deviation reflected greater homogeneity of the postintervention results. The trend toward negative spherical equivalent values occurred due to the total spherical degree, the goal of which is emmetropia with a residual negative cylinder. Entabi et al. presented values similar to those of the present study, with a spherical equivalent ranging from −0.13 ± 2.31 D to −0.26 ± 0.62 D [13].

A search of the literature on PubMed in August 2016 (using the keywords “VFQ 25” and “toric IOL”) did not reveal articles in which the VFQ 25 was applied to patients who had undergone cataract surgery with toric IOL implants. However, greater satisfaction was reported among patients who had received the toric IOL implantation as opposed to those who had received the spheric IOL implantation [14]. These results were consistent with those of the present study, which also found a significant improvement in all of the items evaluated by the questionnaire. It is important to consider the gain in vision quality and quality of life as a result of the simple visual recovery provided by cataract surgery, as demonstrated by other studies in which the questionnaire was applied to patients who had undergone cataract surgery [15, 16]. To et al. reported a variation from 65.19 ± 16.80 points to 88.02 ± 14.51 points in the NEI VFQ 25 of cataract patients who had received surgery for one eye. These results were similar to those of the present study (43.20 ± 15.76 to 79.70 ± 10.11), both with a significant *p* value [15]. Lin et al. presented a mean variation between preoperative and postoperative aspheric and spheric IOLs of 13.95 and 13.02, respectively; the present study found a mean positive variation of 36.49 [16].

More important than refractive, visual, and quality of life data is the interpretation of these findings based on their correlations. The current study revealed statistically significant correlations between patient satisfaction and the visual function items.

When the variation in the NEI VFQ 25 and CS was analyzed, Pearson's coefficient was 0.409 (*p* = 0.033), indicating that the improvement in CS provided by cataract surgery is directly correlated with an increase in visual quality and quality of life.

When variations in BCVA were correlated with the results of the questionnaire, Spearman's correlation coefficient was 0.508 (*p* = 0.009). In addition, when UCVA was correlated with the results of the questionnaire, Pearson's coefficient was 0.548 (*p* = 0.005). This correlation was the highest among the studied items. These findings reflect the importance of refractive results in cataract surgery, particularly in terms of freedom from glasses.

The lack of a statistically significant difference between UCVA of the eye included in the study and the contralateral eye decreased the influence of visual acuity of the contralateral eye as a confounding factor when the benefit of the surgery was analyzed in terms of the results of the quality of life questionnaire.

Thus, according to the results presented here, cataract surgery with a toric IOL implant to achieve better visual and refractive results is associated with a greater patient perception of visual quality and a greater quality of life.

## 5. Conclusion

Patients with low astigmatism who underwent phacoemulsification with a toric IOL implant experienced significant decrease in refractive astigmatism and improvement in their quality of life; this finding positively correlated with variations in CS, UCVA, and BCVA.

## Figures and Tables

**Table 1:** Domains considered in the NEI VFQ-25. The table reports the domains considered in the VFQ-25, the number of items considered in each domain, and their respective questions.

Domain	Number of items	Questions that assessed the domain
General health	1	1
General vision	1	2
Ocular pain	2	4 and 19
Near activities	3	5, 6, and 7
Distance activities	3	8, 9, and 14
*Vision specific*		
Social functioning	2	11 and 13
Mental health	4	3, 21, 22, and 25
Role difficulties	2	17 and 18
Dependency	3	20, 23, and 24
Driving	3	15c, 16, and 16a
Color vision	1	12
Peripheral vision	1	10

**Table 2:** Visual function results. There was a statistically significant difference between the preoperative and postoperative periods when the following visual functions were compared (*p* < 0.001): UCVA, BCVA, and CS.

Function	Preoperative mean	Preoperative SD	Postoperative mean	Postoperative SD	Range of means	*p* value
UCVA (decimal)	<0.20	0.17	0.80	0.19	0.60	<0.001^*α*^
BCVA (decimal)	0.28	0.15	0.97	0.15	0.69	<0.001^*β*^
CS (log)	1.22	0.32	1.74	0.19	0.52	<0.001^*α*^

UCVA = uncorrected visual acuity; BCVA = best-corrected visual acuity; CS = contrast sensitivity.

*α* = paired *t*-test; *β* = Wilcoxon signed-rank test.

**Table 3:** Refraction results. The table shows a statistically significant variation in terms of preoperative and postoperative refractive cylinder values, as well as a lack of statistically significant differences between preoperative and postoperative spherical equivalence.

Field	Preoperative mean	Preoperative SD	Postoperative mean	Postoperative SD	Mean variation	*p* value
Refractive cylinder	−1.23	0.53	−0.34	0.39	0.89	<0.001^*α*^
Spherical equivalence	−0.17	2.33	−0.52	0.35	0.35	0.517

*α* = paired *t*-test.

**Table 4:** Results of the NEI VFQ-25 showing positive variation with statistical significance between preoperative and postoperative values in all fields of the questionnaire and in total score.

Field	Preoperative mean	Preoperative SD	Postoperative mean	Postoperative SD	Mean variation	*p* value
General health	42.85	17.93	63.09	24.52	20.23	0.004^*α*^
General vision	42.85	14.54	72.38	17.50	29.52	<0.001^*α*^
Ocular pain	57.73	26.36	86.90	22.17	29.16	<0.001^*α*^
Near activities	34.52	17.92	77.77	20.12	43.25	<0.001^*α*^
Distance activities	40.07	25.76	84.12	15.11	44.04	<0.001^*α*^
Social functioning	57.73	23.54	92.85	13.44	35.11	<0.001^*α*^
Mental health	35.11	24.16	75.89	22.38	40.77	<0.001^*α*^
Role difficulties	36.30	22.32	84.52	18.91	48.21	<0.001^*α*^
Dependency	54.36	31.36	90.07	18.37	35.71	<0.001^*α*^
Color vision	64.28	29.12	97.61	7.51	33.33	<0.001^*α*^
Peripheral vision	55.95	20.77	88.09	20.33	32.14	<0.001^*α*^
Total	43.20	15.76	79.70	10.11	36.49	<0.001^*α*^

*α* = paired *t*-test.

**Table 5:** Correlations. The table shows the correlation coefficients, all of which were statistically significant between the variations in questionnaire values and UCVA, BCVA, and CS values, which were 0.548, 0.508, and 0.409, respectively. This result demonstrates a direct and positive association between the items evaluated.

Field correlated with the questionnaire	Correlation	*p* value
UCVA	0.548^*γ*^	0.005
BCVA	0.508^*δ*^	0.009
CS	0.409^*γ*^	0.033

UCVA = uncorrected visual acuity; BCVA = best-corrected visual acuity; CS = contrast sensitivity.

*γ* = Pearson's correlation coefficient; *δ* = Spearman's correlation coefficient.
